# How Far Has Repetitive Transcranial Magnetic Stimulation Come Along in Treating Patients With Treatment-Resistant Depression?

**DOI:** 10.7759/cureus.25928

**Published:** 2022-06-14

**Authors:** Jake Vogel, Varun Soti

**Affiliations:** 1 Psychiatry, Lake Erie College of Osteopathic Medicine, Elmira, USA; 2 Pharmacology and Therapeutics, Lake Erie College of Osteopathic Medicine, Elmira, USA

**Keywords:** bilateral stimulation, prefrontal cortex, repetitive transcranial magnetic stimulation, treatment-resistant depression, major depressive disorder (mdd)

## Abstract

Antidepressant drugs have been the mainstay for treating patients with major depressive disorder. However, with a rapid rise in the rates of major depressive disorder, there has been a substantial increase in the resistance to antidepressants in the last decade. This has augmented the need for alternative treatment modalities, including repetitive transcranial magnetic stimulation. This review assesses the progress repetitive transcranial magnetic stimulation has made in treating patients resistant to antidepressants. We conducted a comprehensive literature search following the Preferred Reporting Items for Systematic Reviews and Meta-Analyses guidelines. The clinical studies reviewed under the scope of this paper showed significant benefits in treatment-resistant patients. Several studies demonstrated that the prefrontal cortex's unilateral and bilateral transcranial magnetic stimulation increased the remission rates in active treatment groups compared to the control. Treatments ranged from 10 to 20 sessions, with 1,600 pulses to a maximum of 4,000 pulses in unilateral stimulation and 720 to 2,100 pulses in bilateral stimulation per session. Interestingly, bilateral stimulation utilizing fewer pulses showed notable improvement than a higher number of pulses in unilateral stimulation. However, the lack of standardized dose, dosing frequency, treatment duration, and follow-up protocols warrant further research to bring this therapy into clinical practice.

## Introduction and background

Major depressive disorder (MDD) is described in the Diagnostic and Statistical Manual of Mental Disorders (DSM) as a period of at least two weeks when a person experiences a depressed mood most of the day, nearly every day, or loss of interest or pleasure in daily activities. MDD is one of the largest health care problems worldwide regarding illness-induced disability [1]. From 2010 to 2018, data showed adults with MDD increased from 15.5 million to 17.8 million in the United States of America (USA), a 12.9% increase. As of 2020, an estimated 21 million adults in the USA alone had at least one major depressive episode. This accounts for over 8% of the entire adult population in the USA. The highest portion of these 21 million adults ranging from 18 to 25 years old, with females having a higher prevalence than males [2]. While the rates of adults in the USA with MDD have been increasing, the economic burden of MDD has increased astronomically. From 2010 to 2018, the economic burden of adults with MDD increased by nearly 38%, from $236.6 billion to $326.2 billion [3]. In 2012 alone, the total burden of MDD exceeded the societal burden of cancer ($131 billion) and diabetes ($173 billion) [4]. The economic burden associated with MDD comes in part from the treatment methods involved in managing MDD patients. An estimated 12-month prevalence of medication-treated MDD was 8.9 million adults [5]. Unfortunately, the treatment rates over the past two decades have become stagnant, with 44% of individuals suffering from MDD not accessing related health care services, and many of those who do, are not responding to treatment [6].

While the difficulty accessing appropriate healthcare is a significant problem, another crucial problem individuals with MDD face is resistance to their treatment. Of the estimated 8.9 million adults receiving medication to treat MDD, an estimated 2.8 million had treatment resistance to their medication. This accounts for nearly 31% of adults [5]. With this issue of individuals with MDD becoming resistant to treatment with medications, a different treatment modality becomes necessary to help all those suffering from treatment-resistant depression (TRD).

A promising candidate for treating TRD patients is repetitive transcranial magnetic stimulation (rTMS). Recent studies have evaluated rTMS therapy in treating TRD patients. This review provides an overview of TRD, rTMS, and extensively highlights the effectiveness of using rTMS in TRD patients.

## Review

Literature search and study selection

**Figure 1:**
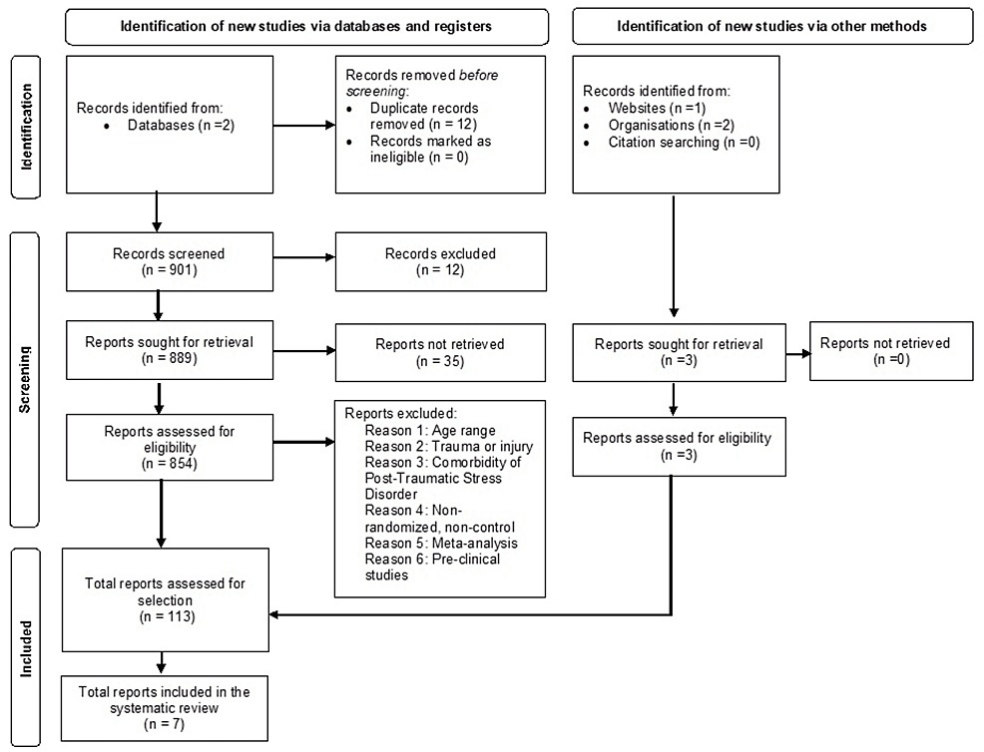
Literature search and study selection. This review utilized PubMed and Clinicaltrials.gov to search for clinical studies on rTMS in treating TRD patients. The keywords were limited to “Transcranial Magnetic Stimulation Depression” and “Treatment-Resistant Depression.” By using filters and inclusion criteria, including articles written in the English language, and complete clinical studies with select patient demographics (male and female, 18-65 years old), the number of studies was narrowed down to 7. n, number; rTMS, repetitive transcranial magnetic stimulation; TRD, treatment-resistant depression.

We conducted a literature search between October 2021 and March 2022 following Preferred Reporting Items for Systematic Reviews and Meta-Analyses guidelines [7]. PubMed and Clinicaltrials.gov were utilized, and only articles written in the English language were selected (Figure 1). The keywords “Transcranial Magnetic Stimulation Depression” yielded 671 results and “Transcranial Magnetic Stimulation Depression and Treatment-Resistant Depression” yielded 191 results on PubMed. On Clinicaltrials.gov, “Transcranial Magnetic Stimulation and Treatment-Resistant Depression” showed 39 studies. Only relevant studies were included and assigned a level of clinical evidence as per the previous literature [8].

Treatment-resistant depression

According to the United States Agency for Healthcare Research and Quality, TRD is defined as depression that fails to respond to a minimum of two antidepressant treatments administered at an adequate dose and duration [4]. Researchers have categorized five stages of TRD: Stage zero is those who have not had a single adequate medication trial; Stage I is a failure of adequate trials of one class of antidepressants; Stage II is a failure of adequate trials of two distinctly different classes of antidepressants; Stage III consists failure of adequate trials of two distinctly different classes of antidepressants and failure to respond to one augmentation strategy, such as lithium or thyroid augmentation; Stage IV consists of all the factors of Stage III and includes the failure to respond to a second augmentation strategy such as monoamine oxidase inhibitors; Stage V has all the aspects of Stage IV along with the failure of an adequate course of electroconvulsive therapy. Along with the stages of TRD, there are several other factors can lead to TRD, including not staying on medicine long enough, skipping doses, and genetics [9].

Left dorsolateral prefrontal cortex and MDD

Past research on depression and the brain using positron emission tomography has shown that in patients with MDD, the left prefrontal cortex exhibited glucose hypometabolism [10]. The prefrontal cortex consists of a dorsolateral and orbitofrontal prefrontal cortex. Therefore, the left dorsolateral prefrontal cortex is of particular interest regarding MDD as it has deep connections to other areas of the brain heavily involved in mood regulation and has also been underactive in patients with MDD. These findings make the left dorsolateral prefrontal cortex an area of profound interest when conducting studies on MDD in general and especially with rTMS [11].

Repetitive transcranial magnetic stimulation

rTMS is a non-invasive neuromodulation technique that involves the application of a magnetic field to a specific area of the brain [5]. The magnetic pulses delivered to the left dorsolateral prefrontal cortex depolarize pyramidal neurons, leading to neurophysiological transsynaptic alterations [12]. One of the mechanisms in which rTMS works is through alterations in gamma-aminobutyric acid (GABA)-ergic neurotransmission, with rTMS, increasing GABA in the medial prefrontal cortex in responders [12]. When using rTMS, several factors are adjusted when designing a treatment protocol, including the frequency, the number of pulses per session, and the length and the time between sessions [10]. Of particular importance is the frequency used when treating patients with MDD. Two types of frequencies utilized are low frequency, 1 Hertz (Hz) or less, and high frequency, 5 Hz or higher. Low and high frequency have their own specific effect, with low frequency inducing inhibitory and high frequency causing excitatory effects [10].

Effectiveness of rTMS in TRD patients

Over the years, researchers have investigated rTMS in treating TRD patients (Table 1). One of the research groups, Avery et al., set out to assess the clinical efficacy of rTMS in treating TRD through a large, double-blind sham-controlled study. They enrolled subjects between the ages of 21 and 65 years old, those who met the DSM IV criteria, failed to respond to or tolerate at least two previous adequate antidepressant trials, and had a score of 17 or greater on the Hamilton Depression Rating Scale (HAMD). Of 91 participants initially screened, only 68 met the criteria and chose to be a part of this study. Of the 68 participants, 35 were randomly selected into the active treatment group and 33 into the sham control group. Participants were also encouraged, though not required, to discontinue any current antidepressant medications they were taking for at least two weeks before the first rTMS session [13].

**Table 1:** Summary of key clinical studies evaluating the rTMS effectiveness in TRD patients. The clinical studies meeting the inclusion criteria of this review showed significant benefits of rTMS in TRD patients. HAMD, MADRS, and SF-36 questionnaires were utilized to assess the patient improvement across these investigations. HAMD, Hamilton depression rating scale; HLF-rTMS, High left frequency repetitive transcranial magnetic stimulation; LFR-rTMS, Low frequency right repetitive transcranial magnetic stimulation; MADRS, Montgomery-Asberg depression rating scale; p, Probability value; rTMS, Repetitive transcranial magnetic stimulation;SF-36, Study-36 item short form; TRD, Treatment-resistant depression.

Authors	Type of study	Level of evidence	Sample size	P-value	Findings
George et al. (2010) [11]	Prospective, multisite, randomized, active sham controlled	I	190 patients	Proportion of remitters p = 0.02 , HAMD p = 0.06, MADRS p = 0.01	rTMS was significant in proportion of remittance (14% compared to 5%) and reducing MADRS scores (29.48 to 24.59).
Godfrey et al. (2021) [12]	Open label study	II.1	27 patients	p < 0.001	rTMS significantly lowered MADRS scores compared to sham (32.7 to 10.7).
Avery et al. (2006) [13]	Double-blind, sham controlled	I	88 patients	Response rate p = 0.008, Remission rate p = 0.033	rTMS had higher response rates (30.6% to 6.1%) and remission rates (20% to 3%) compared to sham.
Fitzgerald et al. (2003) [14]	Double-blind, placebo-controlled	I	60 patients	MADRS scores after two weeks between treatment and sham p = 0.004, MADRS scores after four weeks between HFL-TMS and LFR-TMS p = 0.05	rTMS significantly lowered MADRS scores after two weeks compared to sham control. Bilateral rTMS had significantly lower MADRS scores between weeks two to four.
Fitzgerald et al. (2006) [15]	Double-blinded, randomized controlled trial	I	50 patients	p = 0.005	rTMS significantly lowered the mean MADRS score compared to sham (7.7 compared to 3.2).
Solvason et al. (2014) [16]	Multicentered, randomized, sham controlled	I	301 patients	Mental component score at 4 weeks p = 0.025 and at 6 weeks p = 0.043	rTMS significantly improved SF-36 subscale scores of general health, mental health, and mental component score.
Blumberger et al. (2016) [17]	Double-blind, placebo-controlled	I	121 patients	p = 0.027	Bilateral remission rates were significantly higher than in the sham group.

Along with voluntary discontinuation of antidepressant medication, before the first rTMS session, participants completed the HAMD and Beck Depression Inventory (BDI). HAMD and BDI scores were measured after visits five, 10, 15, and one week after the last rTMS session, and those who had a decrease of 50% or greater in their HAMD score from baseline were reassessed two weeks after the final rTMS session. The first rTMS consisted of 10 Hz applied over the left dorsolateral prefrontal cortex for 1,600 pulses. The course of the study was a total of 15 sessions over four weeks. Study participants were considered either responders or nonresponders. Participants were considered responders if they experienced a decrease of 50% or more significant in their HAMD score. Participants could also be considered a remitter if they had a HAMD score below eight after the one-week post rTMS visit that persisted for two weeks after the last rTMS session. This study reported that the rTMS group had a statistically significant greater response rate (30.6% [11 out of 35 participants]) compared to the sham (6.1% [two out of 33 participants], p = 0.008) [13].

Along with more responders, the rTMS group also had a significantly greater remission rate of 20.0% (seven out of 35 participants) compared to 3.0% (one out of 33 participants) in the sham (p = 0.033). In addition, there were significant findings in the time by group interaction in the rTMS group compared to the sham. These results indicated that the groups had different patterns of change from the first session to the week following the final session. For the HAMD scores, the time by group interaction showed a mean decrease in score of 7.8 compared to 3.7 in the sham (p = 0.002). For the BDI scores, the time by group interaction showed a mean decrease in score of 11.3 in comparison to 4.8 in the sham (p = 0.003). While these results are remarkable, their significance may be impacted due to the relatively small sample size (n = 68) [13].

George et al. also assessed the rTMS effectiveness in treating patients with TRD. They set up a prospective, multisite, randomized, active sham-controlled clinical trial. It consisted of 190 antidepressant-free patients (intention to treat population) with diagnosed MDD and was structured into three phases. Phase I randomized the 190 patients into an active treatment group (n = 92) and a sham (n = 98). The active group received rTMS of 10 Hz over the left dorsolateral prefrontal cortex totaling 3000 pulses per session for 15 sessions over three weeks. If the patient showed a reduction of 30% or more profound in their HAMD score, they continued treatment for up to three additional weeks with HAMD assessments performed twice weekly. However, patients who showed a reduction in HAMD score less than 30% from their baseline score were discontinued and crossed over to phase II. Phase II was an open-label study with a lower number of daily stimuli for unresponsive patients to the phase I treatment. They would continue with phase II for an additional three weeks. Those who improved but had not remitted continued to receive treatment during phase II if they showed progressive improvement with a reduction of at least two points from the HAMD score at every rating. Phase III began when patients met the stable remission criteria, a HAMD score of three or less or two consecutive HAMD scores less than 10 during phase I, the rTMS treatment was tapered off in three weeks, and an antidepressant medication was started [11].

The primary analysis of remission results in the intention to treat population (n = 190) found a significant effect of rTMS treatment compared to sham. Of the 92 patients in the active group, 13 achieved remission (14%) in comparison to the sham, wherein five of the 98 patients achieved remission (5%) (p = 0.02). These results showed that the odds of attaining remission were 4.2 times greater with active rTMS than with sham. Of the 13 remitters in the active group, six achieved remission during weeks one through three of phase I, two achieved remission during day 2 of week 4, three during day 5 of week 4, and two during day 2 of week 5. Compared to the end of phase I, HAMD scores collected as a baseline were not significant (p = 0.06), with a decrease in the active rTMS group from 26.26 to 21.61 compared to a reduction from 26.51 to 23.38 in the sham. The Montgomery Asberg Depression Rating Scale (MADRS) scores showed statistical significance when comparing baseline scores to scores taken at the end of phase I (p = 0.01). MADRS scores in the active rTMS group decreased from 29.48 to 24.59 compared to a decrease from 29.81 to 27.75 seen in the sham [11].

Although this trial yielded significant results, a factor that may impact their relevance is that the patients were selected from the different sites. For example, of the 18 remitters, 15 (83.3%) had come from only two of the four total sites where the patients were selected. Similarly, 81 of the 122 patients who had less treatment resistance (66.4%) were also from only two of the four sites [11].

Interested in understanding how rTMS could work in TRD patients, Godfrey et al. hypothesized that rTMS alters GABA and glutamate levels. To test their hypothesis, they conducted an open-label study on patients between 18 and 65 years with a primary diagnosis of MDD or bipolar disorder, a score of greater than 20 on MADRS, and who failed to respond to two adequate courses of different classes of antidepressants. The study had 31 participants. However, the data of only 27 participants were used for the final analysis. The study duration was four weeks. A frequency of 10 Hz was administered over 20 total sessions with 4,000 pulses per session. Participants were classified as either responders or nonresponders based upon whether they had a reduction in MADRS score of at least 50%. Of 27 participants, 11 were classified as responders, and 16 were considered nonresponders [12].

Among all 27 participants, the mean reduction in MADRS score between baseline to post-rTMS treatment was 30.7 to 19.4 (p < 0.001). The 11 responders experienced a mean reduction of MADRS score of -66.8% from (baseline: 32.7) to (post-TMS: 10.7). However, the 16 nonresponders experienced a mean reduction of MADRS score of -13.5% from 29.3 to 25.4. While the overall reduction in mean MADRS score following rTMS was significant, several factors may have impacted the statistical significance of these findings. For example, there was a lack of a sham control group to compare the efficacy of rTMS. The study relied on using the MADRS score before the start of rTMS treatment as baseline scores instead of using an actual sham control as a baseline group to compare the rTMS efficacy. The other limiting factor can be the relatively small sample size. A sample size of 27, with approximately 12.9% unavailable may impact the study findings’ significance [12].

Interestingly, in another study, Fitzgerald et al. evaluated the efficacy of high-frequency left rTMS (HFL-rTMS) and low-frequency right rTMS (LFR-rTMS) in patients with TRD. They compared the efficacy of HFL-rTMS and LFR-rTMS against a sham-treated control. All 60 patients in this study had to score greater than 20 on the MADRS and must have failed at least two previous antidepressant treatments. Patients were divided into three groups: HFL-rTMS (n=20), LFR-rTMS (n=20), and sham control (n=20). This study compared measurements of the MADRS scores taken at the beginning of the study, after two and four weeks, among the three groups [14].

Patients in the HFL-rTMS group received 1000 pulses at 10 Hz for five days a week over two weeks, whereas patients in the LFR-rTMS group received only 300 pulses at one Hz for five days a week over two weeks. The sham control group patients received sham stimulation five days a week over two weeks. After the initial 10 treatment sessions, patients with a greater than 20% improvement in MADRS score in the active group continued with rTMS for an additional 10 sessions, while patients who did not achieve significant improvement were offered the option to cross over to the other active treatment group. Comparing MADRS scores from the baseline to the scores taken after 10 sessions showed a significant difference between the two treatment groups (13.55 ± 16.7%) compared to changes in the sham (0.76% ± 16.2%) (p = 0.004). However, after four weeks, there was a remarkable improvement in the LFR-rTMS group (38.8% ± 19.7%) in comparison to the HFL-rTMS group (14.1% ± 21.5%) from weeks 2 to 4 [14].

Furthermore, this study would then randomize 11 patients who had initially received sham treatment into active treatment groups for at least ten sessions (seven HFL-rTMS and four LFR-rTMS). Among those 11 patients, there was a mean improvement in the MADRS scores in the LFR-rTMS group of 15.0 ± 10.4 points (45.3%) compared to 0.3 ± 4.1 points (1.3%) improvement in the HFL-rTMS group. However, the sample size was relatively small (N = 60), which may impact the statistical significance of these findings. Another possible potential for bias is an issue with blinding, with 48% of patients correctly guessing their type of treatment before disclosure, 17 of the 40 in the active treatment groups, and 12 of 20 in the sham control group [14].

After establishing the effectiveness of LFR-rTMS over HFL-rTMS in treating patients with TRD, Fitzgerald et al. then assessed the effectiveness of combining LFR-TMS and HFL-TMS in a “bilateral” rTMS treatment in such patients. In a double-blind, randomized controlled trial, they enrolled 50 patients with TRD (25 in the active treatment group, 25 in the sham control group) with a MADRS score greater than 20. Treatment consisted of LFR-rTMS (420 pulses) followed by HFL-rTMS (300 pulses) amounting to 720 pulses per session. Each patient received ten treatment sessions over the course of two weeks. Following the first ten sessions, the initial assessment revealed a mean improvement of the MADRS scores of 7.7 in the active treatment group contrasting to 3.2 in the sham (p < 0.001). To continue participating in the study, patients needed to achieve a further reduction of their MADRS score of at least 10% in the following weekly assessments. After the first two weeks, 15 of the 25 in the active group continued with the study, with only 11 patients completing the full six weeks. However, only seven of 25 continued past two weeks, with no patients progressing past the sixth week. By the end of the study, 11 patients in the active group (25%) met the clinical response (greater than 50% reduction in MADRS score) in comparison to two patients (8%) in the sham (p < 0.005). In addition to these findings, nine patients in the active treatment group met the remission criteria (final MADRS score less than 10) compared to no patients in the sham (p = 0.005). Moreover, of the 18 patients initially in the sham group and then transferred to the active group receiving treatment for at least two weeks, five were able to make it to the sixth week of the trial. This cohort’s mean improvement in MADRS was 37% (p < 0.005), with eight patients meeting clinical response and six the remission criteria. However, this clinical trial had relatively fewer participants (n = 50) than their previous study [15].

Drawing from the conclusions of these studies showcasing the effectiveness of rTMS in treating patients with TRD, other researchers built further to develop an understanding of the positive impact of rTMS in such patients. A group of researchers, notably Solvason et al., sought to study the rTMS effect on the left prefrontal cortex in patients with TRD and further evaluated if rTMS had a positive impact and how long these effects lasted [16].

Solvason et al. enrolled 301 medication-free patients with TRD in a six-week randomized controlled trial. The active group consisted of 155 patients who received 10 Hz of active rTMS for a total of 3,000 pulses per session. The sham control group had 146 patients. Following the initial six-week randomized controlled blinded trial, patients who did not meet the clinical criteria for partial response (a less than 25% reduction in baseline HAMD score) were eligible to enroll in a follow-up six-week open trial. In the open-label phase of this study, 158 patients were enrolled, with 85 and 73 having had received a sham and active rTMS treatment previously, respectively. A total of 120 patients received a clinical benefit from either the randomized controlled blinded trial or the open-label study. Of the 120 patients, 99 (82.5%) benefited from active rTMS and 21 (17.5%) benefited from sham [16].

Following the open-label phase of the study, there was a three-week period before the 24-week durability study where the 120 patients had tapered off their prior rTMS treatment and started on an antidepressant medication monotherapy. Various medications were used by patients, with the most selected drugs being duloxetine, venlafaxine, bupropion, and escitalopram. After the three-week tapering period, the 24-week durability study began. To further measure the effectiveness of treatment, its positive impact, and duration, the Medical Outcomes Study 36-Item Short Form (SF-36) and the Quality-of-Life Enjoyment and Satisfaction Questionnaire (Q-LES-Q) were utilized. In the SF-36, 100 is the maximum score. A score of 50 or above is considered normal. However, any score under 50 represents a deviation from “normal” health and functioning. The Q-LES-Q is a self-administered quality-of-life instrument used to identify the overall level of satisfaction across 14 different aspects of life. Q-LES-Q scores are based on a scale from one to five, with one being very poor and five being very good [16].

SF-36 and Q-LES-Q were measured at the beginning of the study to obtain baseline scores, at weeks four and six during the randomized controlled blinded trial, the open-label phase, and at the end of the 24-week durability study. The study findings demonstrated that patients who received active rTMS, in comparison to sham, in the initial randomized controlled blinded trial showed statistically significant improvements in Q-LES-Q and SF-36 scores at four weeks in the categories of general health (p = 0.049), mental health (p = 0.006), and mental component score (p = 0.025). Also, at six weeks statistically significant improvements in Q-LES-Q and SF-36 scores were seen in general health (p = 0.047), role emotional health (p = 0.044), mental health (p = 0.015), and mental component score (p = 0.043) [16].

In the open-label phase of the study, statistically significant improvements in Q-LES-Q and SF-36 scores for both the previously active and previously sham groups were seen in total score (p < 0.001 for both groups), the role of physical health (p = 0.009 for the previous active group, p = 0.015 for the previous sham group), general health (p < 0.001 both groups), social functioning (p < 0.001 for both groups), the role of emotional health (p = 0.005 for the previous active group, p < 0.001 for the previous sham group), and mental component score (p < 0.001 both groups) at four weeks. At the sixth week mark, statistically significant improvements in Q-LES-Q and SF-36 scores for both the previously active and previously sham groups were seen in total score (p < 0.001 both groups), general health (p < 0.001 both groups), social functioning (p < 0.001 for both groups), the role of emotional health (p < 0.001 both groups), and mental component score (p < 0.001 both groups) [16].

However, the results of the 24-week durability study revealed no significant difference in the Q-LES-L score taken before the 24-week durability study and after (p = 0.124 for the previous active group, p = 0.165 for the previous sham group) and in the SF-36 scores taken before the 24-week durability study and after in the role of physical health (p = 0.215 for the previous active group, p = 0.072 for the previous sham group), general health (p = 0.680 for the previous active group, p = 0.651 for the previous sham group), social functioning (p = 0.613 for the previous active group, p = 0.645 for the previous sham group), the role of emotional health (p = 0.606 for the previous active group, p = 0.120 for the previous sham group), and mental component score (p = 0.936 for the previous active group, p = 0.627 for the previous sham group) [16]. These encouraging results demonstrate the clinical benefits of rTMS in treating patients with TRD in the short term and as a possible long-term treatment option in combination with antidepressant medications. However, there can be skepticism about the open-label arm of the study. This study phase could have led to bias in the patients who had not previously received benefits from rTMS. Also, several different classes of antidepressant medications were used in the study, including selective serotonin and norepinephrine reuptake inhibitors (duloxetine and venlafaxine), and selective serotonin reuptake inhibitors (escitalopram), and norepinephrine-dopamine reuptake inhibitors (bupropion) that could skew the study findings.

Nevertheless, researchers in the last two decades have consistently demonstrated the effectiveness of the prefrontal cortex stimulation using rTMS in patients with TRD. To assess patient improvement, multiple studies used different measurement instruments, including MADRS, HAMD, BDI, Q-LES-Q, and SF-36. While these tools are established and influential in determining depression, a lack of uniformity across clinical studies makes it harder to generalize these findings and use them as a benchmark for clinical practice. Moreover, there is no standardized protocol for the number of pulses per session and the total number of sessions. For example, the number of sessions varied from 10 to 20, and the number of pulses ranged from 1,600 pulses to 4,000 pulses in unilateral stimulation and 720 pulses to 2,100 pulses in bilateral stimulation each session across studies highlighted in this review (Table 2). Therefore, more research is needed to establish the clinical practice of rTMS in treating TRD patients. This can be achieved by standardizing crucial components of rTMS therapy, such as study designs, dosing frequency, number of pulses per session, treatment duration, and scaling scores.

**Table 2:** Comparison of differentiating variables among studies. The clinical studies meeting the selection criteria reviewed in this paper varied from one another in several criteria, including the number of failed trials of antidepressant medications used among patients, number of treatment sessions, number of pulses per session, and the tools used to measure the effectiveness of the specific treatments. HAMD, Hamilton depression rating scale; MADRS, Montgomery-Asberg depression rating scale; p, Probability value, Q-LES-Q, Quality of life enjoyment and satisfaction questionnaire.

Authors	Sample Size	Average number of failed antidepressants trials	Hertz	Number of treatment sessions	Number of pulses	Evaluation method	P-value
George et al. (2010) [11]	190 patients	3 to 6	10	15	3000	HAMD, MADRS	p = 0.06, p = 0.01
Godfrey et al. (2021) [12]	27 patients	4.5	10	20	4000	MADRS	p < 0.001
Avery et al. (2006) [13]	88 patients	2	10	15	1600	HAMD	p = 0.008
Fitzgerald et al. (2003) [14]	60 patients	2	10	10	300 (right) 1000 (left)	MADRS	p = 0.004, p = 0.05
Fitzgerald et al. (2006) [15]	50 patients	2	1 (low) 10 (high)	10	420 (right) 300 (left)	MADRS	p < 0.001, p < 0.001
Solvason et al. (2014) [16]	301 patients	5.5	10		3000	Q-LES-Q	*Blind phase:* General health p = 0.049, Mental component p = 0.025; *Open label phase:* General health p < 0.001, Emotional health p = 0.005, Mental component p < 0.001; *24-week durability phase:* General health p = 0.680, Emotional health p = 0.606, Mental component p = 0.936
Blumberger et al. (2016) [17]	121 patients	2	10	15	600 (low) 1500 (high)	HAMD	p = 0.014, p = 0.20

## Conclusions

MDD continues to be a growing health crisis, with some of the most widely used modern methods becoming ineffective at a staggering pace. With the rates of TRD increasing to new levels over the past decade, the answer to this crisis may lie in alternative methods. Researchers have attempted to address TRD by exploring distinct ways to treat these patients, for example, rTMS. This review highlights the effectiveness of rTMS in treating patients with TRD, showcasing several studies that show the possibility of significant benefits that rTMS could have in treating those suffering from TRD. The most effective is the bilateral rTMS. However, more research is required with larger sample sizes to truly test the effectiveness of rTMS in TRD patients. In addition, more endeavors must be made to further establish the effectiveness of both unilateral HFL-rTMS and bilateral rTMS. The future of psychiatric research in managing patients with TRD continues to be paved with new findings on the science of depression, and treatment modalities such as rTMS have certainly gained wider acknowledgment and come a long way.
